# Case Report of Portal Vein and Inferior Mesenteric Vein Pylephlebitis as Complication of Sigmoid Diverticulitis

**DOI:** 10.3390/reports8020068

**Published:** 2025-05-15

**Authors:** Thomas Ferenc, Vinko Bubić, Tomica Bratić, Vitorio Perić, Ivan Antun Mašić, Vid Vrčić, Filip Ferega, Vinko Vidjak

**Affiliations:** 1 Department of Diagnostic and Interventional Radiology, Merkur University Hospital, 10000 Zagreb, Croatiatomica.bratic@gmail.com (T.B.); vitorioperic@gmail.com (V.P.); vinko.vidjak@gmail.com (V.V.); 2 School of Medicine, University of Zagreb, 10000 Zagreb, Croatia; antun.masic05@gmail.com (I.A.M.); ferega.filip@gmail.com (F.F.)

**Keywords:** pylephlebitis, diverticulitis, sigmoid colon, thrombosis, ultrasound, computed tomography, case report

## Abstract

**Background and Clinical Significance**: Pylephlebitis is a suppurative thrombophlebitis of porto-mesenteric veins. It is a rare complication of intraabdominal infection or inflammation. **Case Presentation**: A 46-year-old female patient presented to the Emergency Department (ED) with a three-day history of subfebrile body temperature (37.5 °C) and dull pain in the right lower abdominal quadrant propagating to the left lower quadrant, with frequent bowel movements and liquid stool consistency. Inflammatory markers were elevated. Following transabdominal ultrasound, possible diagnoses were inflammatory changes of the appendix or sigmoid colon. She was given oral antibiotics and discharged home with a surgical follow-up the next morning. The next day, due to the worsening of the symptoms, surgery was performed with no additional imaging studies. Intraoperative findings were diverticulitis of the sigmoid colon with perforation and peritoneal inflammation, and primary anastomosis with a diverting ileosotomy was performed. The patient was discharged from the hospital after seven days with completed antibiotic treatment. Twelve days later, the patient presented to the ED with a two-day fever (38 °C), elevated inflammatory markers and imaging findings consistent with pylephlebitis: complete left portal vein thrombosis, partial thrombosis of the segmental branch of the right portal vein and thrombosis of the inferior mesenteric vein. The administration of anticoagulants and antibiotics started and after nine days she was discharged home. **Conclusions**: Timely treatment is a necessity in patients with diverticulitis to prevent complications. Furthermore, clinicians and radiologists should be familiar with vascular complications of diverticulitis because their detection and the following treatment can prevent more extensive disease.

## 1. Introduction and Clinical Significance

Pylephlebitis is defined as suppurative thrombophlebitis of the portal and mesenteric veins and is an uncommon complication of intraabdominal/pelvic infection or inflammation [1]. It is often associated with diverticulitis, appendicitis, pancreatitis or cholangitis, cholecystitis, and inflammatory bowel disease [1,2,3,4]. This condition is mainly a sequela of bacteremia in the porto-mesenteric venous system [1]. Additional risk factors are a hypercoagulable state and clotting factor deficiencies [3].

Transabdominal ultrasound (US) with Doppler analysis and contrast-enhanced computed tomography (CT) are the most used imaging studies for detecting pylephlebitis [5]. Treatment is often based on broad-spectrum antibiotics and anticoagulant therapy [1,3], while the primary source of infection can also be treated surgically. The mortality rate is 8.7–19% [1,2,3,5].

Herein, we report a case of a female patient who was surgically treated for perforated sigmoid colon diverticulitis and developed pylephlebitis of the main portal veins and inferior mesenteric vein.

## 2. Case Presentation

A 46-year-old female patient presented to the emergency department (ED) with a three-day history of dull pain in the right lower abdominal quadrant propagating to the left lower abdominal quadrant. She was subfebrile (37.5 °C) with frequent bowel movements and liquid stool consistency (more than four per day in that period). No blood or mucus was detected in the stool, and no significant weight loss was observed in the past three months. The patient’s medical records showed five episodes of recurrent diverticulitides in the last six years, and she was not taking any medications. During clinical examination, the patient complained about dull pain in the right lower abdominal quadrant, particularly during deep abdominal palpation. Laboratory blood tests displayed leukocytosis [16.42 (3.4–9.7*10^9^/L)] with neutrophil predomination [12.78 (2.06–6.49 × 10^9^/L)] and elevated CRP levels [94.3 (<5 mg/L)]. Other blood parameters were within normal reference values.

The attending surgeon referred the patient to the transabdominal US under suspicion of appendicitis or fluid collection. The US demonstrated a tubular structure with an edematous wall and a diameter of 12 mm in the right lower abdominal quadrant, which could be tracked to the left lower abdominal quadrant. The reported possible diagnoses were inflammatory changes of the appendix or sigmoid colon. No free fluid or collection was found. The patient was discharged home with oral antibiotic treatment (ciprofloxacin 500 mg, 2 × 1, for 7 days) and a surgical follow-up the next morning. The next day, due to the worsening of the symptoms, abdominal guarding during clinical examination, and a progressive increase in leukocytosis (19.56 × 10^9^/L), neutrophil (14.78 × 10^9^/L) and CRP levels (194 mg/L), surgical intervention was needed, and no additional imaging studies were performed. Laparoscopy was the intended surgical approach, but it was decided to convert to laparotomy due to findings of diverticulitis of the sigmoid colon with signs of perforation and peritoneal inflammation. Furthermore, the Douglas pouch was filled with purulent content, and no signs of appendiceal or adnexal inflammation were found. The sigmoid colon was resected, and the primary anastomosis with diverting ileosotomy was formed. The pathohistological analysis confirmed the surgical findings with no evidence of tumor cells. After seven days, the patient was discharged from the hospital with the completed intravenous administration of the antibiotic treatment (ciprofloxacin and metronidazole) and regression of inflammatory markers (only CRP levels were elevated −77.7 mg/L).

**Figure 1:**
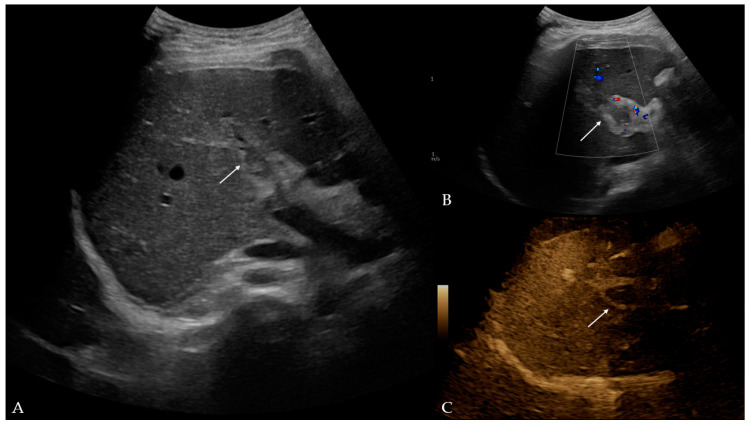
Ultrasound and color Doppler of liver with addition of B-flow option: (**A**) B-mode—hyperechogenic material in lumen of left portal branch, potentially indicative of thrombus (arrow); (**B**) color Doppler—no Doppler signal in left portal branch, which corresponds to thrombus (arrow); (**C**) B-flow—no flow in left portal vein, which confirmed earlier findings (arrow).

**Figure 2:**
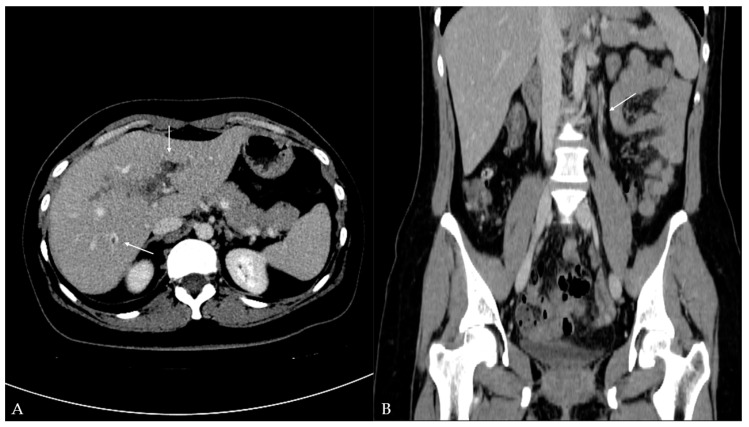
Contrast-enhanced computed tomography of the abdomen and pelvis: (**A**) axial plane—hypodense filling defects (thrombus) in the left portal vein and additionally in the segmental branch of the right portal vein (arrow); (**B**) coronal plane—additional hypodense filling defects (thrombus) detected in the inferior mesenteric vein (arrow).

Twelve days later, the patient presented to the ED with a two-day fever (38 °C) and no other symptoms. The postoperative scar and ileostomy were unremarkable, and no signs of peritonitis were detected on clinical examination. Laboratory blood tests showed leukocytosis [15.56 (3.4–9.7 × 10^9^/L)] with neutrophil predomination [10.99 (2.06–6.49 × 10^9^/L)] and elevated liver enzyme [AST 63 (11–34 U/L), ALT 153 (8–41 U/L), GGT 220 (9–35 U/L), ALP 185 (54–119 U/L)] and CRP levels [99.6 (<5 mg/L)]. Other parameters were within the normal values. The attending surgeon referred the patient to the transabdominal US under suspicion of fluid collection. The US and color Doppler (CD) analysis showed left portal vein thrombosis and preserved patency of the hepatic arteries, veins, and inferior vena cava (Figure 1). No free fluid or collection was found. The patient was then referred to contrast-enhanced abdominal and pelvic CT to evaluate the extent of the thrombosis (Figure 2). The left branch of the portal vein was occluded entirely, and the segmental branch of the right portal vein was partially occluded. Thrombotic content was also found in the inferior mesenteric vein. No free fluid, collection, or liver and bowel perfusion abnormalities were found. Due to the described findings and recent diverticulitis, pylephlebitis was the imaging diagnosis. The patient was admitted to the hospital and put on low-molecular-weight heparin (LMWH) and intravenous administration of antibiotics (meropenem).

Tests for hereditary coagulation disorders showed that the patient is a heterozygote for MTHFR (A1298C) and PAI-1 (4G/5G). Furthermore, she experienced no previous thromboses and had a negative family history of thromboses. The conclusion was that an inflammatory condition provoked the current condition. After nine days, the patient was discharged from the hospital with a prescription of oral antibiotics (cefixime 400 mg, 1 × 1, and metronidazole 400 mg, 3 × 1, for 14 days, respectively) and anticoagulant treatment (dabigatran, 150 mg, 2 × 1, planned for six months). On the first follow-up examination two weeks after discharge, surgical status was satisfactory with normal functioning of the ileostomy, liver enzymes were still elevated [AST 108 (11–34 U/L), ALT 233 (8–41 U/L), GGT 217 (9–35 U/L), ALP 172 (54–119 U/L)], and CRP, thrombocyte and PT/INR levels were within the normal range (<5 mg/L, 318 × 10^9^/L, and 1.0, respectively). The US/CD of the liver showed incipient recanalization of the left portal vein. Four months after the initial diagnosis, the patient is under regular interval monitoring (monthly blood analysis; surgical and US/CD evaluation) and ongoing anticoagulant treatment with progressive recanalization of the thrombosed portal and inferior mesenteric veins.

## 3. Discussion

Pylephlebitis is a rare complication of intraabdominal/pelvic infective or inflammatory process. Choudhry et al. state it was first described in 1846 by Waller [3], while Fusaro et al. state it was Osler in 1882 [5]. The two recently published systematic reviews reported a low incidence rate of this condition (0.37–2.7 cases per 100,000 person-years) [1,5]. A retrospective study by Belhassen-García et al. [4] analyzed 7796 patients admitted to the hospital diagnosed with intraabdominal infections, and only 0.16% of individuals developed pylephlebitis. The number of documented cases has progressively increased in the last 50+ years, and since 1971, around 220 cases have been reported [5]. The median age of patients with pylephlebitis is 49–50 years, with male predominance (70.5–71.8%) [1,5]. The youngest documented patient with this condition was 20 days old [5].

In this case, diverticulitis is considered the leading cause of pylephlebitis. The most common sources of infection are diverticulitis (26.5–30%) and appendicitis (19–22%) [1,2,5]; however, one study revealed pancreatitis as a leading cause of pylephlebitis [3]. Besides sigmoid colon diverticulitis, other sites of diverticulitis have also been reported to cause pylephlebitis: jejunal [6], ileal [7], cecal [8], and ascending colon diverticulitis [9]. Other sources of infection are cholangitis [1,4,5], cholecystitis [4,5], inflammatory bowel disease [2,10], hepatic abscess [1,5], and colovenous fistula [11]. This condition is monomicrobial in 37.9–42.8% and polymicrobial in 24.3–27.2% of cases [1,5], whereas 23–70% of blood cultures are positive [1,2,3,4,5]. According to two recent systematic reviews, the primary causative pathogens were *Escherichia coli* (20.4–25%), *Bacteroides* spp. (12.6–17%), *Streptococcus* spp. (11.7–15%), and *Fusobacterium* spp. (9.7%) [1,5]. In our patient, no blood cultures were taken; therefore, it is unknown what the causative pathogens of this condition were.

The first time our patient presented to the ED with pain in the right lower abdominal quadrant propagating to the left lower abdominal quadrant and elevated body temperature, whereas the second time, she presented only with fever. The patients usually present with symptoms such as fever (75.5–86.4%) and abdominal pain (66.4–82%) [1,2,5]. Clinical signs include right upper quadrant or diffuse abdominal tenderness, hepatomegaly, and splenomegaly [5], whereas sepsis can occur in approximately 58% of patients [1]. Pylephlebitis is considered acute if the symptoms develop within 60 days before hospital admission without evidence of portosystemic collaterals or cavernous transformation of the portal vein [2]. Blood laboratory analysis usually reveals leukocytosis (80–89.7%), elevated C-reactive protein (90.9%), and liver enzyme levels (69–71.6% of cases) [5]. Only 11% of individuals test positive for hereditary coagulation disorders [5]. In a Belhassen-García et al. [4] study, one patient was heterozygous for factor V Leiden and hyperhomocysteinemia, and two were heterozygous for MTHFR. Our patient was heterozygous for MTHFR (A1298C) and PAI-1 (4G/5G).

The transabdominal US combined with Doppler evaluation can detect echogenic material in the portal venous system, disrupting normal blood flow. The US was used in 38.8% of patients to assess this condition [5]. As a superior imaging modality, contrast-enhanced CT is a mainstay for the imaging diagnosis of pylephlebitis and was used in 89.3% of patients [5]. However, the sensitivity and specificity of CT in the diagnosis of pylephlebitis are not known [12,13,14]. Magnetic resonance imaging (MRI) is not routinely used, only for specific indications [5]. US/CD and CT were used for imaging diagnosis in this case, with detection of complete left portal vein thrombosis, partial thrombosis of the segmental branch of the right portal vein, and thrombosis of the inferior mesenteric vein. Thrombi are frequently detected in the main portal vein and/or right or left portal branch (57.3%, 29.1%, 24.3%, respectively), superior and inferior mesenteric vein (38–42%, 2–9.7%, respectively), and splenic vein (12–12.6%) [1,2,5]. In a study by Kanellopoulou et al. [2], intraportal gas was reported in 18% of cases.

The treatment of pylephlebitis is mainly based on a combination of antibiotics, with a mean duration of 25.9 days [1,5]. Suggested antibiotic regimens in pylephlebitis are summarized in a systematic review by Fusaro et al. [5]. If the hepatic abscess is present, it is advised to take antibiotic treatment for up to 4–6 weeks [3]. Although there are no recommendations for prescribing anticoagulants in patients with pylephlebitis, they are often administered (up to 82%) with a mean duration of 128.7 days [1,3,5]. The mortality rate for pylephlebitis is 8.7–19% [1,2,3,5]. There was no statistically significant association between the thrombosis site and the disease outcome; however, independent risk factors for mortality were pertinent comorbidities, positive blood cultures, and sepsis [1].

The study’s retrospective nature and the lack of CT imaging before the surgical intervention and/or before the first hospital discharge are limiting factors. Therefore, the possibility that pylephlebitis existed during that first hospitalization cannot be excluded.

## 4. Conclusions

We reported a female patient who initially presented with sigmoid colon diverticulitis and a medical history of recurrent diverticulitides. Subsequently, she ended up with perforated diverticula, peritoneal inflammation, and a loop ileostomy. Twelve days post-surgery, she presented with imaging findings of thrombotic changes in the left and right portal and inferior mesenteric vein, consistent with pylephlebitis. On the first follow-up examination after discharge, surgical status was satisfactory, with the ileostomy functioning normally, elevated liver enzymes, and CRP levels within the normal range. The US/CD of the liver showed incipient recanalization of the left portal vein. The patient is under regular interval monitoring. Timely treatment is a necessity in patients with diverticulitis to prevent complications. Furthermore, clinicians and radiologists should be familiar with the vascular complications of diverticulitis because their detection and follow-up treatment can prevent more extensive disease.

## Data Availability

Data are contained within the article, and further inquiries can be directed to the corresponding author.

## References

[1] Jevtic D., Gavrancic T., Pantic I., Nordin T., Nordstrom C.W., Antic M., Pantic N., Kaljevic M., Joksimovic B., Jovanovic M. (2022). Suppurative Thrombosis of the Portal Vein (Pylephlebits): A Systematic Review of Literature. J. Clin. Med..

[2] Kanellopoulou T., Alexopoulou A., Theodossiades G., Koskinas J., Archimandritis A.J. (2010). Pylephlebitis: An overview of non-cirrhotic cases and factors related to outcome. Scand. J. Infect. Dis..

[3] Choudhry A.J., Baghdadi Y.M., Amr M.A., Alzghari M.J., Jenkins D.H., Zielinski M.D. (2016). Pylephlebitis: A Review of 95 Cases. J. Gastrointest. Surg..

[4] Belhassen-García M., Gomez-Munuera M., Pardo-Lledias J., Velasco-Tirado V., Perez-Persona E., Galindo-Perez I., Alvela-Suárez L., Romero-Alegría A., Muñoz-Bellvis L., Cordero-Sánchez M. (2014). Pylephlebitis: Incidence and prognosis in a tertiary hospital. Enfermedades Infecc. Y Microbiol. Clín..

[5] Fusaro L., Di Bella S., Martingano P., Crocè L.S., Giuffrè M. (2023). Pylephlebitis: A Systematic Review on Etiology, Diagnosis, and Treatment of Infective Portal Vein Thrombosis. Diagnostics.

[6] Bockmeyer J., Taha-Mehlitz S., Heeren N., Ristic S., Metzger J., Gass J.M. (2020). Jejunal Diverticulosis Probably Leading to Pylephlebitis of the Superior Mesenteric Vein. Case Rep. Surg..

[7] El Mouhadi S., Ait-Oufella H., Maury E., Menu Y., Arrivé L. (2012). Ileal diverticulitis complicated by portal-mesenteric pylephlebitis and pulmonary septic foci. Diagn. Interv. Imaging.

[8] Lee B.K., Ryu H.H. (2012). A case of pylephlebitis secondary to cecal diverticulitis. J. Emerg. Med..

[9] Fukahori M., Shirayama S., Kawasaki A., Takasugi T., Sano H., Iwasaki H. (2015). A case of silent perforated diverticulitis in the ascending colon combined with pylephlebitis resulting in complete occlusion of the portal trunk. Clin. J. Gastroenterol..

[10] Baddley J.W., Singh D., Correa P., Persich N.J. (1999). Crohn’s disease presenting as septic thrombophlebitis of the portal vein (pylephlebitis): Case report and review of the literature. Am. J. Gastroenterol..

[11] Krzak A.M., Townson A., Malam Y., Mathews J. (2022). Diverticulitis complicated by colovenous fistula formation and pylephlebitis. J. Surg. Case Rep..

[12] Chang Y.S., Min S.Y., Joo S.H., Lee S.H. (2008). Septic thrombophlebitis of the porto-mesenteric veins as a complication of acute appendicitis. World J. Gastroenterol..

[13] Wong K., Weisman D.S., Patrice K.A. (2013). Pylephlebitis: A rare complication of an intra-abdominal infection. J. Community Hosp. Intern. Med. Perspect..

[14] Balthazar E.J., Gollapudi P. (2000). Septic thrombophlebitis of the mesenteric and portal veins: CT imaging. J. Comput. Assist. Tomogr..

